# Game of clones: decipher lineage plasticity in hormone-driven cancers

**DOI:** 10.1007/s00018-026-06171-8

**Published:** 2026-04-22

**Authors:** Amanda Leonita, Siyuan Cheng, Joshua Warrick, Isaac Yi Kim, Su Deng, Ping Mu

**Affiliations:** 1 Department of Urology, Yale University School of Medicine, New Haven, CT 06511 USA; 2 Department of Pathology, Yale University School of Medicine, New Haven, CT 06511 USA; 3 Yale Cancer Center, Yale University School of Medicine, New Haven, CT 06511 USA

**Keywords:** Lineage plasticity, Hormone-dependent cancers, Regulatory modules, Multi-omics approaches, Artificial intelligence

## Abstract

Hormone-dependent cancers such as prostate, breast, and endometrial carcinomas rely on nuclear hormone receptors to sustain lineage identity and growth. Therapies targeting androgen, estrogen, or progesterone signaling are initially effective but ultimately impose selective pressures that drive resistance through lineage plasticity, the ability of tumor cells to abandon their native identity and adopt alternative cellular fates. While the biological consequences of lineage plasticity are increasingly recognized, a major challenge lies in defining the regulatory programs that govern these cell fate transitions and determine their stability, directionality, and therapeutic vulnerability. In this review, we focus on the regulatory modules that underlie lineage plasticity in hormone-driven cancers. We highlight how integrative multi-omics approaches spanning genomic, transcriptomic, epigenomic, proteomic, and chromatin-level layers have enabled the identification of transcriptional and epigenetic programs that destabilize lineage fidelity. We discuss how single-cell and spatial technologies have revealed intermediate states, rare subpopulations, and microenvironmental influences that shape plasticity trajectories. Finally, we emphasize the role of artificial intelligence and machine learning as integrative tools to reconstruct gene regulatory circuits, infer fate transitions, and connect molecular programs to phenotypic outcomes. By synthesizing these biological insights across experimental and computational modalities, we propose a conceptual framework for understanding lineage plasticity in hormone-driven cancers, with emphasis on prostate and breast malignancies. This integrated perspective highlights how regulatory programs governing cell identity can be revealed, perturbed, and potentially constrained, offering opportunities to identify biomarkers, expose therapeutic vulnerabilities, and ultimately translate mechanistic understanding into strategies that limit resistance.

## Introduction

Hormone-dependent cancers, including prostate, breast, and endometrial carcinomas, are some of the most common and clinically significant malignancies worldwide [90]. These tumors originate from hormone-responsive epithelial tissues that rely on nuclear hormone receptors to drive lineage-specific gene expression programs that support growth, differentiation, and survival. As such hormone signaling through receptors, such as the androgen receptor (AR), estrogen receptor alpha (ERa), and progesterone receptor (PR), represents a central therapeutic vulnerability. In prostate cancer, standard-of-care involves utilizing androgen deprivation therapy (ADT) and next-generation AR-targeted therapies to suppress androgen signaling [95, 99]. Breast cancer treatments frequently employ endocrine therapies such as selective estrogen receptor modulators (SERMs), aromatase inhibitors, or selective estrogen receptor degraders (SERDs) [80].

Historically, resistance to targeted cancer therapies has been understood through a genetic lens driven by the acquisition or selection of mutations, copy number alterations (CNA), or pathway reactivation events that restore oncogenic signaling [83, 84]. However, these mechanisms alone cannot explain the rapid emergence of resistance and phenotypic heterogeneity observed in patients or the reversibility of resistant states in cancer cells. Lineage plasticity has therefore gained increasing attention as a model of resistance that incorporates genetic alterations that permissively destabilize lineage identity while emphasizing non-genetic transcriptional and epigenetic reprogramming as active drivers of therapy adaptation [38, 39]. Throughout this review, we define cell state as the dynamic and potentially reversible molecular configuration of a tumor cell, whereas cell fate refers to a stable lineage commitment defined by sustained regulatory circuits. Within this framework, resistance can arise not only through clonal selection of pre-existing resistant populations, but also through adaptive state switching, in which genetically similar tumor cells dynamically transition between phenotypic states under therapeutic pressure [70]. These transitions may involve stable transdifferentiation into alternative lineages or reversible, metastable plastic states that coexist within tumors, conferring adaptive flexibility. Clinically, these therapy-induced identity shifts are associated with aggressive disease, treatment failure, and poor prognosis.

Given the complexity and dynamism of these transcriptional networks, integrative approaches are required to unravel the molecular intricacies of lineage plasticity. Single-omics techniques often fall short of capturing multi-scale regulatory interactions that drive state transitions. By contrast, newer multi-omics strategies which combine transcriptomic, epigenomic, chromatin conformation, and proteomic data, provide a more comprehensive view of regulatory architecture and its context-dependent modulation [74]. Single cell and spatial technologies further enable the resolution of intermediate cell states and microenvironmental influences that contribute to plasticity [27, 82]. In parallel, artificial intelligence (AI) tools are increasingly applied to infer gene regulatory networks, identify key drivers of cell fate transitions, and integrate heterogeneous datasets across time and space [77]. These computational frameworks aid to identify regulatory patterns and predicting how perturbations reshape cell identity.

**Fig. 1:**
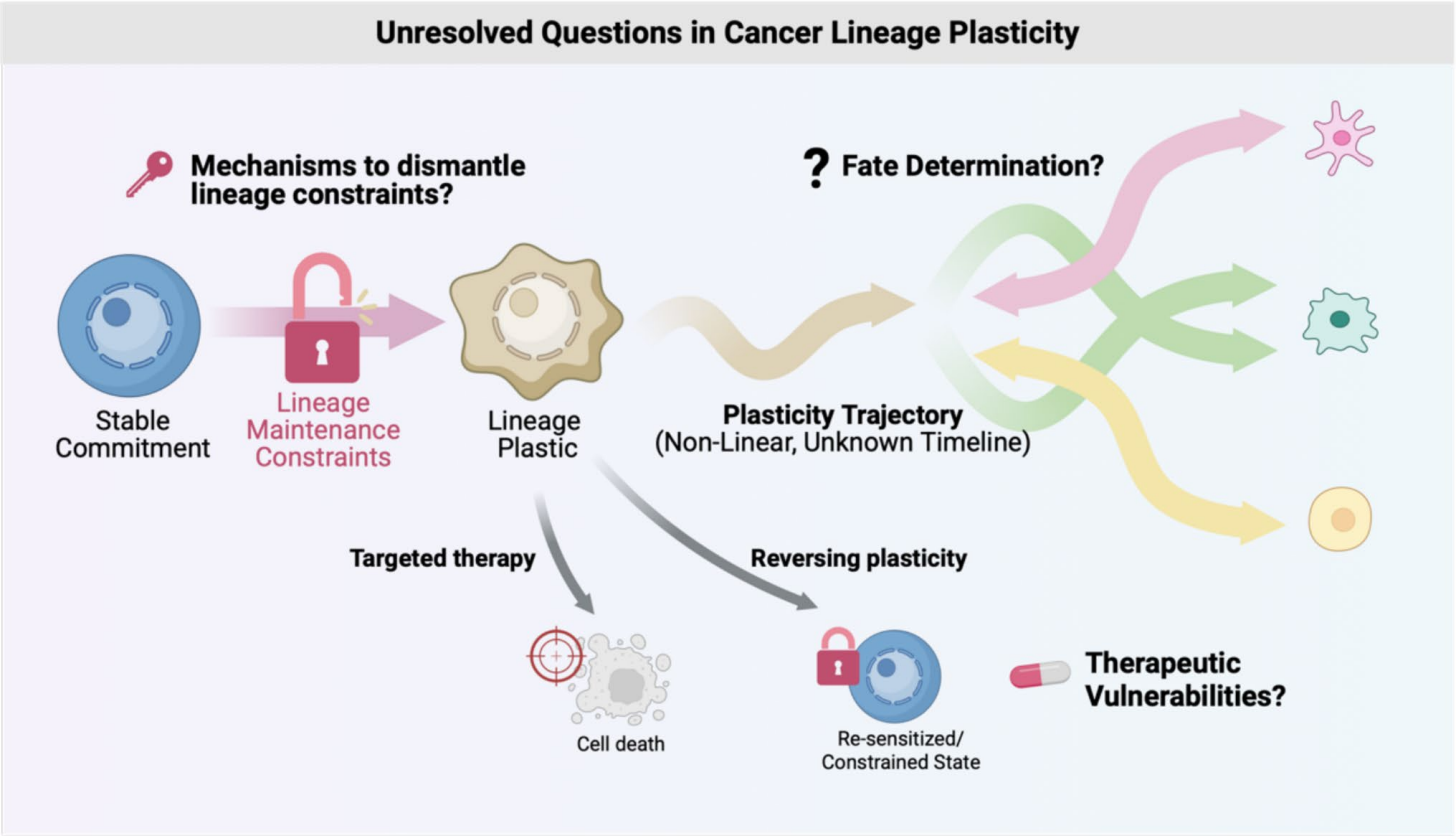
Conceptual framework of key unresolved questions driving cancer lineage plasticity and therapy resistance. This schematic illustrates the transition of tumor cells from a fixed identity to a dynamic, resistant state, highlighting fundamental gaps in current biological understanding. **Mechanisms to dismantle lineage constraints.** Under homeostatic conditions, the lineage maintenance constraints are in place to maintain stable commitment to the cell lineage. Our understanding of regulatory programs that enforce commitment and the mechanisms by which they are dismantled remain incomplete. **Determining lineage trajectories.** Following the loss of lineage constraints, the lineage trajectory plastic cells are dynamic and has unclear timelines. Bidirectional arrows emphasize the potential for reversibility and dynamic transitions between these states. **Identification of therapeutic vulnerabilities.** The entire plasticity process is underpinned by a complex underlying regulatory network that integrate transcriptional control (TFs), chromatin organization, and signaling pathways. A critical unmet need is identifying specific, druggable nodes within this network to identify targeted therapies for lineage plastic cells. Alternatively, we could aim to constrain plasticity and re-sensitize cells to therapy. Figure made by Biorender

Despite growing recognition of lineage plasticity as a driver of resistance, several fundamental biological questions remain unresolved. What regulatory programs actively maintain lineage commitment in hormone-dependent epithelia, and how are these constraints dismantled under therapeutic pressure? What determines whether a tumor cell undergoes transdifferentiation toward a neuroendocrine fate, adopts a stem-like or basal-like state, or occupies an intermediate, metastable identity? Finally, which components of these regulatory networks represent points of vulnerability that can be therapeutically targeted to constrain or reverse plasticity? Addressing these questions requires not only descriptive profiling but an integrated understanding of transcriptional control, chromatin organization, and cell state dynamics. In this review, we synthesize recent biological insights into the regulatory modules that underlie lineage plasticity in hormone-dependent cancers, drawing on advances in multi-omic, single-cell, spatial, and computational approaches. We highlight studies that have used integrative multi-omic and computational approaches to define these modules and link them to therapy resistance, focusing on prostate and breast cancer as primary case studies. We also discuss emerging experimental and AI-based techniques for mapping and perturbing these networks at scale. By examining the interplay between transcriptional regulation, chromatin state, and cell identity, we aim to provide a conceptual and methodological framework for understanding and ultimately targeting lineage plasticity in the clinic, summarized schematically in Fig. 1.

**Fig. 2:**
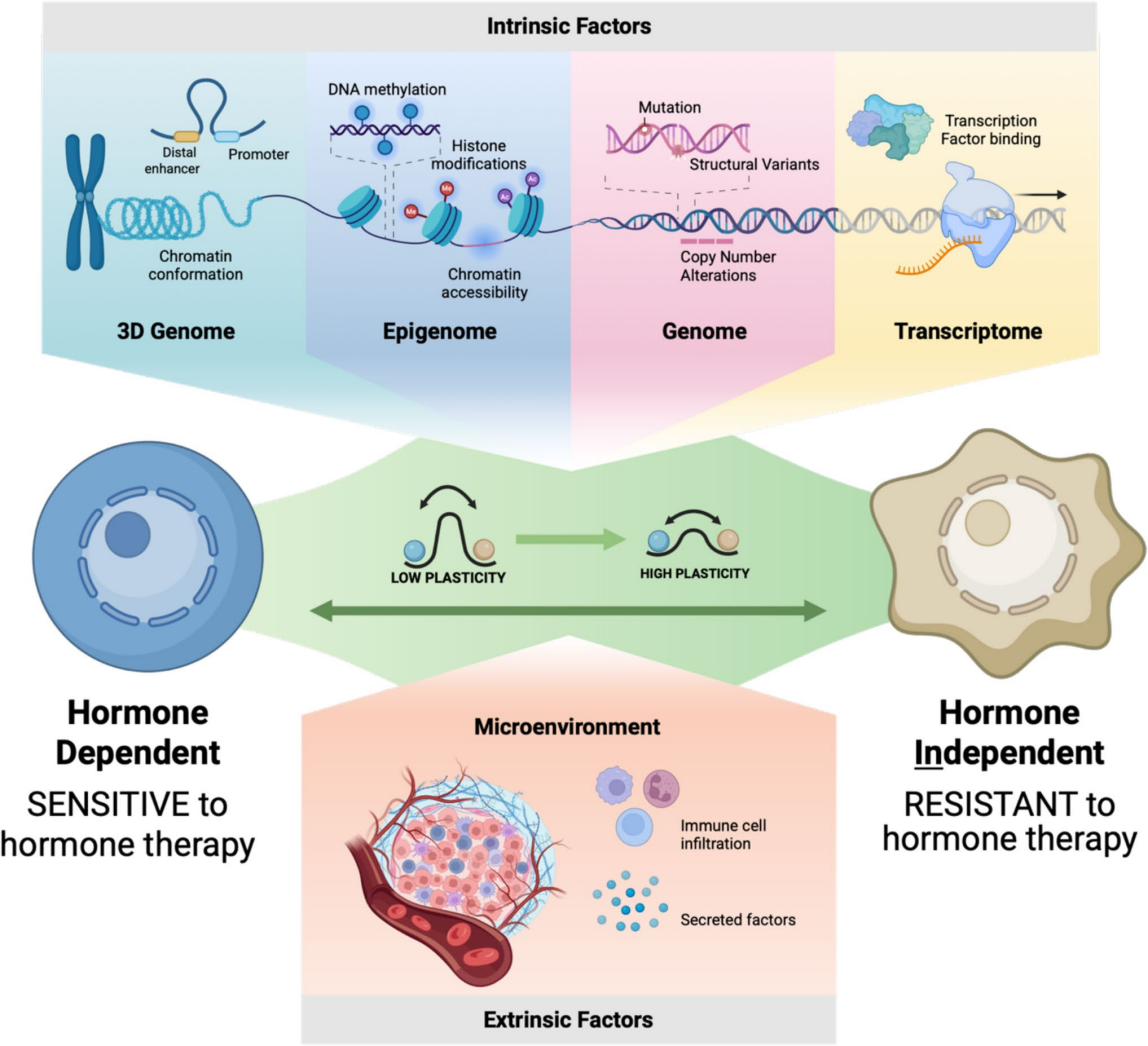
The convergence of intrinsic and extrinsic factors drives lineage plasticity and hormone therapy resistance. The schematic illustrates the interplay of multi-layered regulatory mechanisms that confer plasticity and promote the transition of cancer cells from a hormone therapy-sensitive to a hormone therapy-resistant state. Figure made by Biorender

## Regulatory modules in lineage plasticity

Cellular identity is maintained through tightly coordinated transcriptional programs governed by the interplay of transcription factors (TFs), cis-regulatory elements, and the surrounding chromatin landscape [42]. Lineage plasticity describes a cell’s capacity to undergo a durable shift in identity. This adoption of an alternate/hybrid state can be selected by microenvironmental or therapy-induced stress, thereby enabling survival under pressures that would otherwise be lethal. While differentiation has long been conceptualized as a branching hierarchy, plasticity is better understood as continuous state landscapes [69].

Importantly, lineage plasticity is not inherently pathological but instead represents a fundamental feature of normal tissue biology. In normal tissues, regeneration to restore function after injury may involve transient dedifferentiation or fate flexibility of committed progenitors. For example, intestinal epithelial progenitors can revert to stem-like states after damage to replenish lost stem cells and enable regeneration. This has been demonstrated by lineage-tracing studies showing secretory progenitors regaining stemness and rebuilding crypt architecture [26, 100]. Aberrant plasticity also does not necessarily confer malignancy though it may form premalignant lesions such as with Barrett’s esophagus. Barrett’s esophagus is a premalignant condition in which the normal stratified squamous epithelium of the distal esophagus is replaced by a columnar, intestine-like epithelium in response to chronic gastroesophageal reflux-induced injury. This case of metaplasia has exhibited populations with stable, non-malignant lineage reprogramming that persist for years without inevitable progression to cancer [98, 104, 109].

### Hormone receptors as lineage determinants

Hormone signaling is a core mechanism by which multicellular organisms coordinate development, cellular differentiation, and tissue homeostasis. Hormone-responsive tissues depend on nuclear hormone receptors to regulate cellular proliferation, survival, and lineage specification. These receptors are ligand-activated transcription factors that coordinate tissue-specific gene expression profiles, defining organ development and function [66]. In the mammary epithelium, estrogen receptor alpha (ERα) and progesterone receptor guide ductal morphogenesis, cell cycle progression, and differentiation during normal development [22, 40, 68]. Similarly, the androgen receptor is essential for glandular development, secretory function, and the maintenance of luminal identity in the prostate [91, 102, 105]. Due to this dependence on hormonal controls, epithelial cancers arising from these epithelial cells often retain a dependency on signaling pathways. For instance, most prostate cancers are AR-positive, while 70% of breast cancers express ERα [78].

In hormone-dependent cancers, hormone receptor signaling is implicated in the proliferative drive and contributes fundamentally to sustaining lineage identity. Pioneer factors and lineage-restricting transcriptional co-regulators further determine the specificity of hormone receptors. For instance, FOXA1, a forkhead family pioneer factor, is known to play a pivotal role in enabling chromatin accessibility at hormone response elements. Thus, FOXA1 expression enables ERα binding in breast tissue and AR binding in prostate tissue, resulting in chromosomal regulation implicated in cancer progression [5, 9, 30, 67, 89]. In breast cancer, GATA3 is a crucial factor that collaborates with ERα to maintain a luminal fate and suppress basal or mesenchymal reprogramming [88]. While in the prostate, transcription factors such as NKX3.1 and HOXB13 function downstream of AR signaling to reinforce luminal lineage identity and fine-tune receptor binding specificity [1, 11, 21, 81].

Lineage regulation is also defined at the chromatin level, as hormone receptors cooperate with coactivators and chromatin readers, such as MED1 and BRD4, to establish high-density regulatory domains known as super-enhancers [87]. Super-enhancers act as transcriptional control hubs for lineage-defining and oncogenic genes, such as BRD4, CDK7 and ERG in prostate cancer [19, 43]. These super-enhancers sustain tumorigenic or therapy-resistant transcriptional programs. In castration-resistant prostate cancer (CRPC), loss of AR signaling can lead to changes in the enhancer landscape, resulting in the aberrant expression of genes that define non-luminal, alternative lineages. In breast cancer, plasticity and resistance to endocrine therapy is observed during GATA3 and AP1 mediated super-enhancer remodelling [12, 13].

It is important to note that lineage plasticity is not restricted to hormone receptor–positive cancers undergoing endocrine therapy. Triple-negative breast cancer (TNBC), a subtype defined by the absence of ER, PR, and HER2 expression, can itself be conceptualized as a hormone lineage–abandoned state and therefore represents a parallel example to double-negative prostate cancer (DNPC), which similarly lacks AR signaling and canonical luminal identity programs. TNBC comprises a heterogeneous group of clones characterized by distinct molecular subtypes including epithelial, mesenchymal, and stem-like populations [45, 55]. Functional studies demonstrate that these states are not strictly hierarchical, as sorted subpopulations can re-establish phenotypic equilibrium over time [36], and breast cancer stem-like cells have been shown to transition between epithelial and mesenchymal cell states [60]. In vivo studies with patient-derived TNBC samples further reveal the emergence of reversible drug-tolerant states under chemotherapy, indicating non-genetic, context-dependent plasticity rather than solely clonal selection [25, 71]. These observations suggest that while hormone receptor pathways serve as key stabilizers of luminal identity, the loss or absence of such lineage anchors, as exemplified by both TNBC and DNPC, underscores that lineage plasticity reflects broader gene regulatory network dynamics beyond endocrine therapy–induced suppression.

### Disruptions of lineage fidelity in cancer

Under the selective pressure of therapies targeting receptor signaling pathways, a subset of endocrine-related cancers can escape lineage-defining constraints. This disruption of lineage fidelity has emerged as a hallmark of treatment resistance, involving a complex restructuring of transcriptional and epigenetic programs that define epithelial identity.

**Epithelial-to-Mesenchymal Plasticity (EMP)** is a dynamic and reversible process that allows epithelial cells to acquire mesenchymal traits to promote cell migration [34, 75]. Rather than a binary switch between fully epithelial and fully mesenchymal states, EMP often occurs through intermediate or hybrid epithelial-to-mesenchymal transition (hEMT) states, where individual tumor cells coexpress both epithelial and mesenchymal markers. Moreover, these hEMT states have high metastatic and tumor-initiating potential [50, 79]. Plasticity is further evident in the bidirectional interconversion between mesenchymal-like and epithelial-like breast cancer stem cells, which share transcriptional programs but differ in spatial localization and proliferative behavior [60]. Key transcription factors such as ZEB1, SLUG, and SOX9 actively reprogram luminal progenitor cells and suppress epithelial differentiation, with SOX9 driving luminal-to-basal lineage transitions and promoting basal-like breast cancer progression [35, 49]. These mechanisms highlight how EMP and stemness programs co-opt developmental regulators to erode lineage boundaries and establish plastic states capable of resisting differentiation cues and therapeutic pressure.

**Transdifferentiation** results in cells acquiring tissue-specific traits distinct from the original lineage. This process involves abandoning the lineage the cell is initially committed to and switching to an entirely different developmental program. In prostate cancer, this is indicated by the loss of canonical luminal markers, including KLK3 (PSA), TMPRSS2, NKX3.1, and HOXB13. Instead, tumor cells undergoing lineage plasticity exhibit markers of alternate lineages. For instance, neuroendocrine prostate cancer (NEPC) tumors are characterized by the expression of neuronal lineage markers, such as chromogranin A (CHGA) and synaptophysin (SYP). However, not all prostate cancer lineage plasticity leads to neuroendocrine differentiation. Double negative prostate cancer (DNPC) tumors are a clinically aggressive and therapy resistant variant defined by the absence of both AR activity and neuroendocrine marker expression. DNPC often retain features of epithelial or basal-like identity but exhibit stem-like transcriptional programs and alternative growth signals such as FGF or MAPK pathway activation [16, 21]. Like NEPC, DNPC arises through transcriptional and epigenetic reprogramming rather than genomic mutations, reinforcing the idea that lineage plasticity is largely driven by non-genetic mechanisms of adaptation [93].

## Mapping sequence-to-chromatin landscapes of lineage plasticity

Lineage plasticity emerges when the molecular constraints that stabilize cell identity are disrupted, allowing cancer cells to deviate from lineage-committed transcriptional programs. These constraints are encoded across multiple regulatory layers, spanning DNA sequence, chromatin organization, and transcriptional control. Dissecting how these layers are altered during plasticity is essential for understanding how hormone-dependent cancers escape lineage fidelity under therapeutic pressure.

### Genomic alterations that license lineage plasticity

At the genomic level, lineage plasticity can be facilitated by alterations that weaken lineage-enforcing programs or license alternative cell fates. These include point mutations, copy number alterations, structural rearrangements, and disruptions of regulatory elements that collectively reshape the regulatory potential of the cancer genome. Whole genome sequencing (WGS) and whole exome sequencing (WES) have therefore been foundational for defining the mutational landscape that permits lineage plasticity and therapy resistance. WGS provides a comprehensive analysis of the entire genome, including alterations in non-coding regulatory regions and splice sites, while WES focuses on protein-coding areas, which are more cost-effective but may miss mutations in non-coding regions.

Both WES and WGS have provided insights into the genetic underpinnings of regulatory module dysfunction that enables plasticity and resistance in endocrine-related cancers. In prostate cancer, concurrent loss of tumor suppressors *TP53* and *RB1* are among the most prominent alterations associated with therapy-induced plasticity [51, 72, 83]. These mutations promote independence from AR signaling and promote transdifferentiation towards the neuroendocrine cell fate. Similar mutations in *TP53*, are highly prevalent in breast cancer tumors characterized with stem-like features, EMT activation, and loss of epithelial markers.

Lineage plasticity, however, often arises not from permanent genomic lesions, but through epigenetic and transcriptional reprogramming that cannot be captured by DNA sequencing alone. For example, plasticity-enabling events include mutation or functional loss of SWI/SNF chromatin remodeling components, such as *ARID1A* and *SMARCA4*, which disrupt enhancer–promoter interactions and de-repress developmentally silenced, non-luminal gene programs [23, 103, 106].These alterations reshape the chromatin landscape without altering the DNA sequence, illustrating a fundamental limitation of WES and WGS. In addition to transcriptional and epigenetic regulators, post-transcriptional mechanisms can influence lineage plasticity by shaping the mutational landscape of regulatory networks. Loss of the RNA-binding protein SYNCRIP has been shown to promote APOBEC-driven mutagenesis in prostate cancer, resulting in the accumulation of mutations within key transcriptional regulators, including FOXA1, STAT3, and EP300 [56, 57]. These mutations enhance the activity of lineage plasticity–associated transcriptional programs and facilitate adaptive reprogramming under therapeutic pressure. In this context, SYNCRIP functions as a genomic safeguard that restrains stress-induced mutagenesis, and its loss indirectly activates plasticity-driving transcriptional circuits through mutation-mediated mechanisms. This work establishes a mechanistic link between post-transcriptional regulation, intrinsic mutational processes, and lineage plasticity, highlighting how noncanonical pathways can couple genome instability to cell fate evolution. Thus, while genomic sequencing defines the mutational backdrop that may license plasticity, it is insufficient on its own to decode the multi-layered regulatory networks governing lineage state transitions. Integration of transcriptomic, epigenomic, and chromatin-level data is therefore required to capture the full spectrum of regulatory rewiring that drives phenotypic adaptation.

### Transcriptional rewiring during lineage state transitions

The shift from lineage maintenance to plasticity drivers is orchestrated by the reorganization of lineage-enforcing TFs with plasticity-promoting master regulators. In prostate cancer, combined loss of TP53 and RB1 destabilizes luminal transcriptional programs and upregulates SOX2, a master regulator in maintaining a neural progenitor identity, which drives neuroendocrine reprogramming [7, 32, 52, 72]. This is rarely the work of a single gene, and a global view of transcription reprogramming has been uncovered by bulk RNA sequencing. Upstream regulator analyses reveal that these transitions rely on coordinated modules driven by factors such as EZH2, N-MYC, BRN2, and FOXA1 [15] These factors act cooperatively to repress epithelial signatures while simultaneously activating mesenchymal or neural-like gene sets, effectively rewiring the cell’s internal logic to evade therapeutic pressure [8, 14, 54, 101]. 

Beyond canonical lineage-determining transcription factors, additional transcriptional regulators contribute to adaptive rewiring of lineage programs during therapy resistance. ZNF397 has been identified as a transcriptional modulator that restrains lineage plasticity by maintaining epithelial gene expression programs in prostate cancer [107]. Loss or suppression of ZNF397 leads to derepression of alternative lineage-associated transcriptional networks and increased cellular adaptability under therapeutic stress. Mechanistically, ZNF397 functions as part of a broader transcriptional regulatory layer that shapes lineage trajectories without directly enforcing terminal differentiation. These findings underscore the importance of transcriptional fine-tuning mechanisms in governing plasticity, revealing that lineage instability can arise not only through activation of master regulators but also through erosion of stabilizing transcriptional constraints.

Transcriptional rewiring rarely results in an immediate, binary switch from one distinct cell type to another. Instead, it generates a spectrum of intermediate states characterized by "multilineage" gene expression. Cancer cells can exploit intrinsic regenerative capacity to survive stress. For instance, castration in prostate models triggers a regression to a progenitor-like state, a dormant developmental program used for tissue regeneration. Cancer cells hijack this intrinsic "stemness" program to persist in a drug-tolerant state, indistinguishable from the organ’s natural regenerative pool [46]. Biological interrogation of these heterogeneous populations reveals cells that violate the canonical rules of differentiation, such as the simultaneous co-expression of luminal (epithelial) and neuroendocrine markers, or basal and luminal programs in breast cancer. These hybrid phenotypes represent lineage bifurcation events, where a single cell creates a confusion of identity, possessing the transcriptional machinery to traverse multiple differentiation trajectories simultaneously [10]. Single cell RNA-sequencing (scRNA-seq) has been utilized to identify transcriptional heterogeneity underlying plastic phenotypes in rare subclones and the inference of lineage trajectories from static datasets.

### Epigenetic remodeling of lineage-defining regulatory landscapes

While transcriptomics captures the output of lineage programs, lineage identity is stabilized at the chromatin level through epigenetic constraints that regulate transcription factor access to lineage-defining regulatory elements. Lineage plasticity therefore requires coordinated remodeling of chromatin accessibility and histone modifications to dismantle these constraints and enable alternative transcriptional states. Profiling chromatin accessibility identifies regulatory elements that become permissive during this process and reveals the transcription factors that actively drive lineage switching in cancer cells.

ATAC-seq has become a key tool to map genome-wide chromatin accessibility with high sensitivity and low input requirements. In prostate cancer, ATAC-seq has been used to reveal enhancer reprogramming in therapy-resistant states. For example, Nouruzi et al. [76] found that anti-androgen treatment induces accessibility changes at enhancers associated with neuronal/stem-like differentiation, with increased binding of lineage-reprogramming TFs such as ASCL1 and NEUROD1 [76]. Similarly, in breast cancer, loss of the CoREST complex in endocrine-resistant breast cancer cells leads to reduced accessibility at estrogen signaling regions and increased accessibility at EMT-associated enhancers. ATAC-seq revealed that LSD1, a CoREST component, shifts its binding from FOXA1-associated sites in parental cells to AP-1 and REST motifs in reprogrammed cells, illustrating how CoREST loss reprograms the chromatin landscape during lineage plasticity [31].

Beyond chromatin accessibility, histone modifications provide an essential layer of epigenetic regulation that defines active, poised, or repressed chromatin environments [6]. In the context of lineage plasticity, histone modifications facilitate transitions between transcriptional programs by silencing lineage-enforcing genes and enabling the expression of alternative fate determinants. These marks can be mapped genome-wide using chromatin immunoprecipitation followed by sequencing (ChIP-seq), or with more efficient, lower-input techniques such as CUT&RUN and CUT&Tag [47, 92], which offer improved resolution and signal-to-noise ratios for profiling histone marks in rare or heterogeneous cancer populations.

DNA methylation profiling has revealed distinct epigenetic signatures between adenocarcinoma and NEPC, NEPC tumors display hypermethylation of luminal AR-regulated genes and demethylation of neuroendocrine lineage programs, supporting transcriptional reprogramming [7, 48]. Similarly, in basal-like breast cancers, DNA methylation silences epithelial differentiation genes while promoting mesenchymal and stem-like phenotypes [29]. These epigenetic switches are often orchestrated by mutations or altered expression of DNA methyltransferases (DNMTs) and ten-eleven translocation (TET) enzymes, further reinforcing lineage dysregulation. Importantly, studies have shown that methylation alterations can modulate chromatin accessibility and disrupt insulator function at topologically associating domain (TAD) boundaries, indirectly influencing enhancer–promoter communication and long-range transcriptional control [28]. This contributes to the stochastic activation of oncogenic programs and cell fate transitions.

In prostate cancer, disruption of chromatin organization itself can act as a primary driver of lineage plasticity. Loss of CHD1, a chromatin remodeler enriched at active, lineage-associated regulatory regions, has been shown to profoundly alter nucleosome positioning and enhancer organization, leading to destabilization of androgen receptor–driven transcriptional programs [2, 20, 110, 112, 113]. CHD1 deficiency results in impaired maintenance of luminal identity and increased transcriptional heterogeneity, creating a permissive chromatin environment for alternative lineage programs to emerge [56, 57]. Importantly, these effects occur without directly inducing neuroendocrine differentiation, suggesting that CHD1 loss functions as a lineage gatekeeper whose disruption licenses plasticity rather than dictating a specific fate. This work established chromatin architecture as an active determinant of lineage stability in prostate cancer, highlighting how epigenetic remodeling can precede and enable subsequent transcriptional reprogramming.

### Reorganization of 3D chromatin architecture in lineage switching

Lineage-specific gene expression programs are shaped not only by local regulatory elements but also by higher-order chromatin organization that governs long-range enhancer–promoter interactions. Disruption of this 3D genome architecture can therefore rewire lineage-defining transcriptional networks and promote plastic cell states in cancer. Chromatin conformation capture technologies such as Hi-C, ChIA-PET, and promoter-capture Hi-C have revealed that long-range interactions between enhancers and promoters can be reconfigured during oncogenesis, altering gene regulation at key lineage-specific loci [28, 73].

In hormone-responsive cancers, 3D genome remodeling frequently involves lineage-defining transcription factors. For instance, in breast cancer, enhancer–promoter looping involving the estrogen receptor (ER) and FOXA1 reshapes chromatin topology to sustain luminal gene expression programs [86]. Similarly, androgen receptor (AR)-dependent looping structures in prostate cancer anchor key oncogenic hubs, and disruption of these loops has been associated with transitions to AR-independent states [81]. These reconfigurations often coincide with enhancer hijacking or formation of neo-enhancers that activate alternative lineage regulators such as MYCN and ASCL1 in neuroendocrine prostate cancer [4, 8].

Moreover, chromatin topology changes may reinforce epigenetic plasticity by altering topologically associating domain (TAD) boundaries or by enabling crosstalk between normally insulated genomic regions. Such changes can create ectopic enhancer-gene contacts that bypass normal lineage constraints, supporting transdifferentiation and therapy resistance [28]. The application of Hi-C to patient-derived xenografts and organoids has begun to uncover subtype-specific chromatin loops that correlate with distinct transcriptional states, although their mechanistic contributions to plasticity remain underexplored in prostate and breast cancers specifically. Hi-C analysis revealed that NEPC exhibits distinct 3D chromatin architecture compared to CRPC, with chromatin looping facilitating interactions between enhancer-bound FOXA2 and promoter-bound NKX2.1. NKX2.1 acts as a master regulator of neuroendocrine transdifferentiation by recruiting p300/CBP to remodel chromatin and activate neural lineage programs, highlighting epigenetic and spatial genome reorganization as key drivers of lineage plasticity in prostate cancer [65].

While still an emerging area, integrating 3D genome data with transcriptomic and epigenomic profiles offers the potential to reveal higher-order regulatory logic that underlies lineage switching. However, limitations remain, including the relatively low resolution of current 3D datasets and the need for methods such as single-cell Hi-C to resolve locus-specific interactions within rare or transitioning cell populations.Collectively, these genomic, transcriptional, epigenetic, and chromatin architectural studies have begun to define the key regulatory nodes that govern lineage plasticity in hormone-driven cancers, which are summarized in Table 1.

**Table 1:** Progress in defining key regulatory nodes underlying lineage plasticity in hormone-driven cancers

Regulatory layer/node	Representative factors or mechanisms	Primary evidence types	Key biological insight	Cancer context
Lineage-enforcing transcription factors	AR, ERα, GATA3, NKX3.1, HOXB13	Genomics, transcriptomics, ChIP-seq	Hormone receptors and cooperating transcription factors actively maintain lineage fidelity; suppression destabilizes cell identity	Prostate, breast
Tumor suppressor alterations	TP53, RB1	WES/WGS, functional models	Loss of lineage-restricting checkpoints licenses plasticity and therapy adaptation	Prostate
Plasticity-associated master regulators	SOX2, N-MYC, BRN2, ASCL1	Bulk RNA-seq, scRNA-seq, perturbation studies	Activation drives transdifferentiation or stem-like programs under therapeutic pressure	Prostate
Chromatin remodeling complexes	SWI/SNF (ARID1A, SMARCA4), CoREST	ATAC-seq, ChIP-seq, CUT&RUN	Epigenetic remodeling enables enhancer reprogramming and alternative fate adoption	Prostate, breast
Super-enhancer organization	BRD4, MED1, AP-1–associated assemblies	ChIP-seq, chromatin interaction assays	Redistribution of regulatory hubs supports lineage switching and resistance programs	Prostate, breast
DNA methylation programs	DNMTs, TET enzymes	DNA methylation profiling	Stable silencing of lineage genes and activation of alternative lineage programs	Prostate, breast
3D chromatin architecture	Enhancer–promoter looping, TAD reorganization	Hi-C, ChIA-PET	Spatial genome reorganization reinforces new lineage-specific transcriptional states	Prostate
Intermediate and hybrid cell states	Mixed luminal–neuroendocrine or epithelial–mesenchymal programs	scRNA-seq, spatial transcriptomics	Lineage plasticity proceeds through metastable transitional states rather than binary switches	Prostate, breast
Tumor microenvironmental cues	Immune niches, stromal signaling	Spatial omics, integrative single-cell analyses	Local microenvironmental interactions shape emergence and stabilization of plastic states	Prostate
AI-inferred regulatory modules	Latent factors, gene regulatory networks	Multi-omic AI modeling	Computational integration reconstructs regulatory logic governing lineage transitions	Cross-cancer

## Emerging approaches to identifying regulatory modules of lineage plasticity

Lineage plasticity is not a uniform property of all tumor cells but instead arises from heterogeneous and often rare cellular populations that undergo dynamic state transitions under selective pressure. These plastic states are frequently transient, spatially restricted, and regulated by coordinated changes across multiple molecular layers. Dissecting the regulatory modules that govern these transitions therefore requires approaches capable of resolving cellular heterogeneity, capturing intermediate states, and integrating regulatory information across modalities.

### Resolving cellular heterogeneity and transitional states underlying lineage plasticity

Lineage plasticity arises from heterogeneous tumor cell populations that occupy distinct, intermediate, or metastable lineage states rather than discrete, terminal identities. These transitional states are often rare but biologically decisive, serving as reservoirs for therapy resistance, disease progression, and lineage switching. Resolving such states is therefore essential for understanding how regulatory modules are reconfigured during plasticity. Single-cell transcriptomic and epigenomic approaches enable direct interrogation of these heterogeneous populations at cellular resolution. By profiling gene expression and chromatin accessibility in individual cells, these methods allow reconstruction of regulatory trajectories, identification of lineage-instable subpopulations, and inference of transcription factors and chromatin programs that drive cell state transitions. Increasingly, multi-omic single-cell platforms further link chromatin remodeling to transcriptional output, providing insight into the regulatory hierarchy underlying lineage plasticity.

Integrating multiple single-cell modalities considers layers of gene regulation that underlie lineage plasticity. For instance, scATAC-seq with scRNA-seq using computational tools such as ArchR [33, 97] allows joint inference of TF activity and target gene networks, providing deeper insight into regulatory module dynamics. As changes in chromatin accessibility often precede detectable shifts in gene expression, ATAC-seq offers an approach to identify pioneer factors of lineage plasticity. Thus, integrating chromatin accessibility data with genomic and transcriptomic datasets can elucidate the hierarchical sequence of events driving lineage reprogramming.

Computational frameworks allow joint embedding of datasets to reconstruct cell-state manifolds and infer cross-modality regulatory modules. However, despite advances in multi-omic data integration, several limitations remain. With single-cell omics, data often suffer from sparsity, dropout and technical noise which complicates quantitative comparisons across modalities and patients. Batch effects and variable depth between sequencing platforms limit scalability and reproducibility.

### Spatial regulation of lineage plasticity by the tumor microenvironment

Lineage plasticity is shaped not only by cell-intrinsic regulatory programs but also by spatially localized interactions between tumor cells and their surrounding microenvironment [58, 111], as shown in Fig. 2. Distinct niches within tumors can impose selective pressures that promote lineage instability, foster transitional cell states, or stabilize alternative identities through paracrine signaling, immune interactions, or metabolic stress. Understanding where plasticity emerges within tissue architecture is therefore critical for defining its biological drivers.

Spatial transcriptomic (ST) and imaging-based approaches enable the mapping of transcriptional states and regulatory programs back to their native tissue context [17, 44, 96, 108]. These technologies have revealed that lineage transitions often arise in spatially restricted regions, where rare plastic cells coexist with lineage-committed populations or interface with specific stromal and immune compartments. By integrating spatial information with single-cell profiling, these approaches link regulatory state, cellular identity, and microenvironmental influence, providing a more complete biological framework for understanding lineage plasticity in hormone-driven cancers.

An example is the transformation to ASCL1 + NEPC following RB1 deletion studied by Romero et al. [85]. Using 10X Genomics Visium spatial transcriptomics on prostate tumors with mixed prostate adenocarcinoma and NEPC histology, ST showed that NEPC form spatially discrete regions within adenocarcinoma-dominant tissues. Spatial maps also uncovered small clusters of ASCL1 + cells embedded within luminal (KRT8 +) regions, indicating that neuroendocrine transition begins as rare, spatially localized events [85]. ST using Slide-seqV2 has distinguished progenitor-like/transitional states in tumor and adjacent-normal tissues. This spatial disorganization reflects a loss of stable differentiation boundaries, a phenotype of plasticity. Additionally, EMT-like programs are found to be spatially enriched near tumors. Moreover, spatial proximity analysis resolved myeloid-rich and exhausted-T-cell niches in epithelial cells [41].

Accumulating evidence indicates that inflammatory cytokine signaling within the tumor microenvironment can directly drive lineage plasticity through non-genetic mechanisms. Among these, interferon-gamma (IFNγ) has emerged as a key mediator linking immune pressure to cancer cell fate reprogramming. Independent studies have demonstrated that sustained IFNγ exposure induces transcriptional and epigenetic remodeling programs in prostate cancer cells that promote lineage instability, immune adaptation, and phenotypic evolution, even in the absence of canonical plasticity-associated mutations [18, 24, 61]. These findings establish IFNγ signaling as a microenvironment-driven regulator of lineage plasticity that operates in parallel with, and in some contexts independently from, genetic alterations. Collectively, this work highlights immune-mediated signaling as an underappreciated but potent force shaping cancer lineage trajectories and therapeutic resistance.

Sequencing based ST has primarily been used in studies of cancer plasticity. This technology relies on capturing RNA from spatially barcoded locations on a slide or bead array, followed by NGS readout. Examples of this technology include 10X Genomics Visium HD, Slide-seqV2, and DBiT-seq. Meanwhile, imaging-based ST utilizes in situ hybridization or sequence-by-imaging. This allows detection of RNA molecules within tissue directly using fluorescent probes. Examples include MERFISH, seqFIST, and Xenium in situ. ST can be further integrated with other single cell omics, most commonly with scRNA-seq. Due to the inherent cellular and functional complexity of the tumor microenvironment, no single method will fully resolve its spatial organization and biological diversity. Coupling ST with scRNA-seq for instance allows cell states defined in dissociated data to be mapped back into tissue space, effectively linking molecular identity with spatial context [62].

## Inferring regulatory logic of lineage plasticity with AI-based models

A central biological challenge in lineage plasticity is understanding how regulatory programs enable continuous, reversible, and nonlinear transitions between cell states. These transitions are governed by complex interactions among transcription factors, chromatin accessibility, and cis-regulatory elements, which together define the stability or instability of lineage identity. Artificial intelligence (AI) and machine learning approaches have emerged as powerful tools to integrate high-dimensional genomic and epigenomic data and infer the regulatory logic that underlies these biological processes. Rather than replacing experimental investigation, these models provide a framework for reconstructing regulatory networks, identifying candidate drivers of plasticity, and generating testable biological hypotheses.

### Cis-regulatory sequence determinants of lineage identity

Lineage commitment and plasticity are ultimately encoded within cis-regulatory DNA sequences that determine transcription factor binding, chromatin accessibility, and enhancer activity. Variations in these sequences, particularly within non-coding regulatory regions, can alter the regulatory potential of a cell and bias lineage decisions. AI-based sequence-to-function models have been developed to predict how DNA sequence features shape regulatory activity, enabling the identification of regulatory elements and variants that influence lineage stability and plasticity. These models provide insight into how sequence-level information contributes to the establishment or disruption of lineage-defining transcriptional programs.

Pushing this further, AlphaGenome was recently introduced as a multimodal deep learning model that accepts 1 megabase of DNA sequence and predicts thousands of functional genomic tracks. This can range from chromatin accessibility and histone modifications to splicing and gene expression at single-base resolution [3]. Trained jointly on human and mouse genomes, AlphaGenome accurately predicted the functional effects of genetic variants and recapitulated disease-relevant regulatory mechanisms. For instance, it identified noncoding variants near the TAL1 oncogene that alter chromatin accessibility and transcription factor binding. This type of multimodal prediction is especially relevant in lineage plasticity, where enhancer rewiring and chromatin remodeling are often central to the transition between cellular states [3].

### Mixed, hybrid, and metastable cell states during lineage plasticity

Lineage plasticity frequently manifests not as abrupt transitions between discrete identities, but as mixed, hybrid, or metastable cell states that occupy intermediate positions along a continuum of lineage commitment. These states reflect partial and dynamic reprogramming of regulatory networks, often co-expressing markers of multiple lineages, and are increasingly recognized as biologically important intermediates that precede stable lineage switching, therapeutic resistance, or disease progression. Accurately identifying and characterizing these intermediate states is therefore essential for understanding how lineage plasticity unfolds at the cellular level.

Single-cell transcriptomic and epigenomic profiling has made these transitional states observable, but conventional analysis workflows that rely on deterministic clustering can impose artificial boundaries on inherently continuous processes. AI-based latent variable models, including variational autoencoder frameworks such as scVI, enable representation of cell identity within continuous latent spaces while accounting for batch effects and technical noise [63]. These representations facilitate the detection of rare or intermediate cell states that would otherwise be obscured. Building on this, models such as scGen extend this framework to predict how cells transition between states under perturbations, providing insight into therapy-induced plasticity [64]. In multi-omic settings, integrative approaches such as scMVP jointly model gene expression and chromatin accessibility, enabling reconstruction of shared and modality-specific regulatory programs that underlie mixed and transitional lineage states [59]. This approach is valuable for studying lineage plasticity, as the transcriptional output of a cell often reflects complex changes in chromatin state, enhancer usage, and TF network dynamics.

### Modeling dynamic transitions between lineage fates

Lineage plasticity is fundamentally a dynamic biological process in which tumor cells traverse continuous and often reversible transitions between regulatory states in response to therapeutic and environmental pressures. These transitions do not necessarily follow linear or hierarchical trajectories but can involve branching, convergence, or oscillation between partially committed identities. Accurately modeling these dynamics is critical for understanding when and how lineage commitment is destabilized, and for identifying regulatory tipping points that govern the emergence or stabilization of plastic states.

Single-cell profiling has enabled inference of cell-state dynamics, but commonly used approaches impose assumptions that may not hold in the context of lineage plasticity. RNA velocity infers future transcriptional states by modeling the ratio of unspliced to spliced mRNA, assuming relatively stable transcription, splicing, and degradation kinetics, assumptions that are frequently violated during rapid regulatory reprogramming associated with plasticity [53]. Similarly, pseudotime-based methods arrange cells along a one-directional axis representing progression through a biological process, which is useful for developmental hierarchies but poorly suited to represent multidirectional or reversible transitions characteristic of lineage plasticity [37].

To address these limitations, AI-based latent trajectory models have been developed to learn continuous representations of cell identity together with their underlying dynamics. By coupling latent embeddings with dynamical system frameworks such as neural ordinary differential equations (Neural ODEs), these models enable inference of smooth, nonlinear fate transitions over continuous time [94]. Such approaches provide a biologically meaningful framework for modeling how regulatory programs evolve during lineage plasticity, offering insight into the temporal structure, directionality, and potential reversibility of cell fate transitions rather than static endpoints.

### Future outlook

Understanding lineage plasticity ultimately requires moving beyond static descriptions of cell fates toward predictive models of how regulatory programs evolve under perturbation. A central biological goal is to determine whether plastic transitions are reversible or locked, to identify early regulatory events that precede overt lineage switching, and to define points at which lineage commitment can be reinforced or alternative identities constrained. Addressing these questions demands integrative frameworks capable of linking molecular regulation to dynamic cell fate behavior.

AI-based models are increasingly positioned to support these biological objectives by integrating multi-modal single-cell, epigenomic, spatial, and imaging data into unified representations of cell fate and regulatory logic. As the scale and quality of such datasets continue to expand, these models are expected to improve in their ability to predict how cells respond to genetic or therapeutic perturbations. Importantly, AI-driven predictions are most powerful when coupled to experimental validation, including CRISPR-based perturbation screens, lineage tracing, live-cell imaging, and patient-derived organoid models, which provide causal tests of inferred regulatory mechanisms.

Looking forward, the integration of computational inference with functional experimentation offers a path toward translating regulatory insights into actionable strategies. By identifying regulatory nodes that stabilize plastic fates or gate lineage transitions, AI-informed approaches may help guide combination therapies designed to prevent, delay, or reverse therapy-induced plasticity. In this way, AI serves not as an endpoint, but as an enabling layer that connects regulatory biology to experimental validation and, ultimately, therapeutic intervention. Across these approaches, AI functions as an integrative framework to uncover biological principles governing lineage stability and plasticity, rather than as an end.

## Challenges, future perspectives, and conclusions

Lineage plasticity represents a central biological mechanism through which hormone-dependent cancer cells adapt to therapeutic pressure and evolve aggressive phenotypes. Rather than reflecting a single transdifferentiation event, plasticity arises from coordinated disruption and reorganization of regulatory programs that govern lineage identity. Networks of transcription factors, enhancers, chromatin remodelers, and epigenetic states collectively define the stability of cell identity, and their rewiring underlies the emergence of alternative lineage fates that evade therapy.

Despite major advances in profiling technologies, a key challenge remains distinguishing regulatory drivers of plasticity from downstream or correlative changes. Most datasets capture static snapshots of heterogeneous tumors, making it difficult to infer causality or temporal ordering of regulatory events. Without lineage tracing, time-resolved sampling, or perturbation-based validation, it remains unclear which regulatory changes initiate lineage destabilization, and which merely reflect adaptation to an already altered fate. This limitation is particularly relevant in hormone-driven cancers, where plastic transitions may occur gradually, stochastically, or within rare subpopulations that are difficult to capture.

Another major challenge is that lineage plasticity exists along a continuum rather than as a binary switch between discrete identities. Single-cell analyses across prostate and breast cancers consistently reveal hybrid, intermediate, or metastable states that co-express features of multiple lineages. These states can be transient yet biologically consequential, serving as intermediates toward stable lineage switching or reservoirs of therapeutic resistance. Accurately modeling such continua requires analytical frameworks that move beyond rigid clustering or linear hierarchies to capture multidirectional and potentially reversible fate transitions.

Progress in the field increasingly depends on integrating multi-modal single-cell and spatial profiling with functional interrogation. Technologies that jointly measure transcriptional and chromatin states provide a foundation for identifying candidate regulatory modules, but causal understanding requires perturbation-based approaches such as CRISPR screening, epigenome editing, and lineage tracing. When combined with patient-derived organoid models and in vivo systems, these approaches enable direct testing of how specific regulatory nodes influence lineage stability and plasticity under defined conditions.

Looking forward, the convergence of integrative profiling, computational inference, and functional validation offers a path toward translating insights into clinical impact. Rather than treating lineage plasticity as an inevitable outcome of therapy, a deeper understanding of its regulatory logic may reveal opportunities to constrain or redirect cell fate decisions. Identifying regulatory nodes that stabilize lineage commitment or gate plastic transitions could inform rational combination therapies aimed at preventing or reversing resistance.

In conclusion, lineage plasticity reflects the inherent adaptability of cancer cell identity governed by dynamic regulatory programs rather than fixed genetic lesions alone. Integrative approaches that combine biological insight with single-cell, spatial, and computational frameworks are redefining how these programs are studied. By anchoring technological advances to fundamental biological questions and causal validation, the field is positioned to transform descriptive knowledge of plasticity into actionable strategies that limit tumor evolution and improve therapeutic durability.

## Data Availability

Not applicable.

## Declarations

### Declaration of AI-assisted technologies in the manuscript preparation process

During the preparation of this work the author(s) used ChatGPT in order to proofread and correct grammar and typographical errors. After using this tool/service, the author(s) reviewed and edited the content as needed and take(s) full responsibility for the content of the publication.
